# Pseudoseptic Arthritis: An Initial Presentation of Underlying Psoriatic Arthritis

**DOI:** 10.7759/cureus.14660

**Published:** 2021-04-24

**Authors:** Prodip Paul, Mishouri Paul, Dipon Dey, Julio Ramos, Amit Sharma

**Affiliations:** 1 Internal Medicine, Geisinger Community Medical Center, Scranton, USA; 2 Medicine, Interfaith Medical Center, New York, USA; 3 Epidemiology and Public Health, ZWH Medical Care PC, New York, USA; 4 Rheumatology, Ramos Rheumatology, PC, Scranton, USA; 5 Infectious Disease, Geisinger Community Medical Center, Scranton, USA

**Keywords:** pseudoseptic arthritis, psoriatic arthritis, septic arthritis

## Abstract

Pseudoseptic arthritis is an acute inflammatory monoarthritis that clinically mimics septic arthritis. We encountered an 86-year-old male with a past medical history of hypertension, type 2 diabetes mellitus, and chronic kidney disease (CKD) stage 3 who presented in the emergency department with acute onset of severe left knee joint pain and was started on antibiotics for suspected septic arthritis. Septic arthritis was ruled out with negative synovial fluid culture. Timely initiation of steroids rapidly improved his condition, and he was discharged in stable condition on the 7th day of admission. Though pseudoseptic arthritis has been reported in a variety of settings including rheumatoid arthritis, ankylosing spondylitis, and medication such as intraarticular hyaluronic acid injection, our case report presents a case of pseudoseptic arthritis as the initial presentation of undiagnosed psoriatic arthritis.

## Introduction

Pseudoseptic arthritis is an acute inflammatory monoarthritis. Many varieties of arthritis present in a similar fashion with acute onset of painful swelling and warmth of affected joint with or without fever [1].

As per the case series by Oppermann et al., the most commonly involved joint is the knee joint, while other large joints can also be involved [2]. It is a diagnosis of exclusion and septic arthritis must be ruled out, which is a more serious and potentially fatal condition. Due to similar history, clinical presentation, and serum lab values, synovial fluid Gram stain and culture are required to differentiate pseudoseptic arthritis from septic arthritis [1,2]. Thus, pseudoseptic arthritis is diagnosed after a sterile synovial fluid study. Before the final diagnosis of pseudoseptic arthritis, it is important to start antibiotics. Once the diagnosis of septic arthritis has been ruled out with certainty, antibiotics may be discontinued. Initiation of nonsteroidal anti-inflammatory drugs (NSAIDs) or steroid arrests inflammatory response and leads to rapid improvement [2].

Few potential causes leading to pseudoseptic arthritis include rheumatoid arthritis (RA), gout, pseudogout [2,3], Behcet's disease [4], systemic lupus erythematosus (SLE) [5], ankylosing spondylitis [6], intraarticular hyaluronic acid injection [7], renal transplantation, prosthetic joints, and psoriasis [2]. Though pseudoseptic arthritis is diagnosed after negative synovial fluid culture, each of mentioned potential causes has its unique feature which helps it to distinguish from the other causes. It has also been suggested that some patients with pseudoseptic arthritis are diagnosed with rheumatic disease later on [3].

Pseudoseptic arthritis has been reported as the initial presentation of ankylosing spondylitis [6]. Here, we present a case of pseudoseptic arthritis which initially presented with classic signs and symptoms of septic arthritis of the knee joint but was ultimately diagnosed with pseudoseptic arthritis with underlying psoriatic arthritis.

## Case presentation

An 86-year-old Caucasian male with a past medical history of hypertension, type 2 diabetes mellitus, chronic kidney disease (CKD) stage 3, chronic obstructive pulmonary disease (COPD), and bilateral knee osteoarthritis presented to the emergency department with severe left knee pain, swelling, and warmth for the past four days.

The pain was sharp, 10 out of 10 in intensity (on a scale of 0 to 10, where 0 = no pain and 10 = worst pain), worse with ambulation. In addition to left knee pain, the patient also complained of chronic left elbow and feet pain. On examination, the left knee joint was warm, swollen, and severely tender with markedly restricted range of motion. He mentioned being treated with an intraarticular steroid injection every three months due to his longstanding history of bilateral knee osteoarthritis. His last intra-articular steroid injection was two months back.

**Figure 1:**
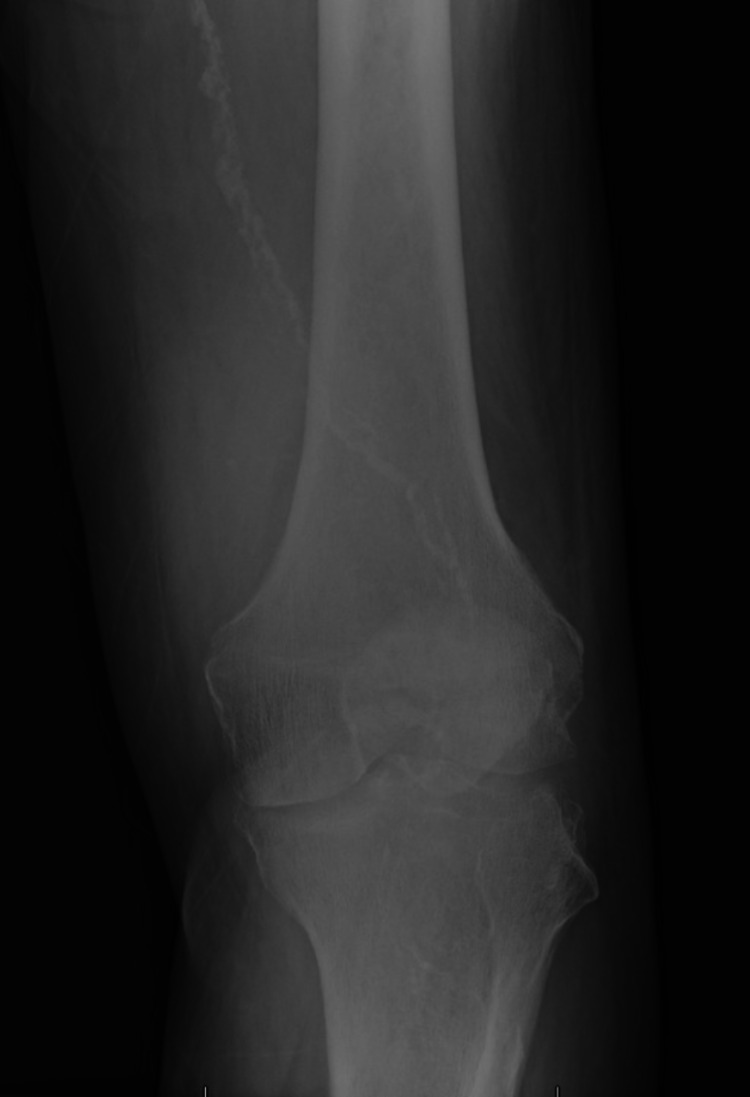
X-ray left knee (A/P view)

**Figure 2:**
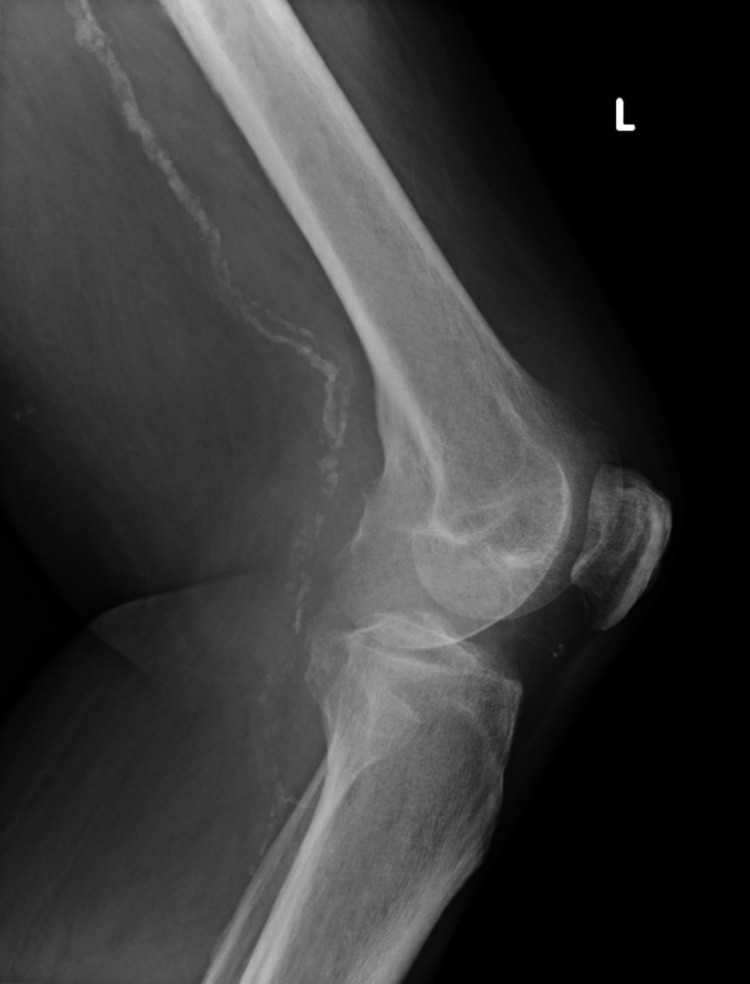
X-ray left knee (Lateral view)

X-ray of the left knee showed severe osteoarthritic changes (Figures 1, 2).

Figure 1 and Figure 2 show severe left medial tibiofemoral compartment osteoarthrosis with osteophyte formation and joint space loss.

Complete blood count (CBC) revealed leucocytosis with white blood cells (WBC) count of 12030 with 77.8% neutrophils, erythrocyte sedimentation rate (ESR) 119, and C-reactive protein (CRP) 217. Based on clinical presentations, physical examination, and abnormal lab findings, septic arthritis was suspected and an arthrocentesis of the left knee was performed. Joint fluid aspiration revealed turbid yellow fluid. After aspiration, joint fluid was sent for further study, gram stain, and culture. The patient was empirically started on IV vancomycin and cefepime for suspected septic arthritis. For further evaluation of presumptive septic arthritis, orthopedic and infectious diseases specialists were also consulted. Synovial fluid analysis revealed significant leukocytosis (WBC count of 89,200) with 100% neutrophil and negative for crystals. During the hospital stay, the patient had persistent fever with no response to antibiotics. The patient was immunocompetent. Despite broad-spectrum antibiotic coverage, the patient’s symptoms did not improve.

After the negative synovial fluid culture, he was suspected to have pseudoseptic arthritis. Blood cultures were negative as well. Since no infectious etiology was found, arthroscopy was not performed. Due to non-resolving arthritis in the context of negative infectious etiology, a Rheumatology opinion was sought. RA factor was positive. Due to the patient's history of colitis, antineutrophil cytoplasmic antibody (ANCA) and anti-saccharomyces cerevisae antibody (ASCA) antibodies were done to rule out enteropathic arthritis and these test results were negative. Finally, the patient was diagnosed with pseudoseptic arthritis of the left knee joint and all antibiotics were discontinued. The patient was given one dose of intravenous solumedrol 40 mg followed by oral prednisone 20 mg twice daily. Steroids not only helped to resolve left knee pain but also improved left elbow pain and bilateral feet pain. NSAID was not prescribed due to underlying CKD. The patient has a family history of psoriatic arthritis and the patient was noted to have multiple skin lesions consistent with psoriatic plaque and onycholysis of bilateral toenails. In view of psoriatic skin lesions, polyarthritis, onycholysis, and positive family history, the patient was diagnosed with psoriatic arthritis. The patient was also started on leflunomide for psoriatic arthritis. The patient was discharged on the 7th day of admission in stable condition with oral prednisone and leflunomide with outpatient Rheumatology follow-up.

## Discussion

Pseudoseptic arthritis is an increasingly recognized entity [1,2]. Despite this, there is limited published literature on the topic. It presents as acute inflammatory monoarthritis with intense pain, effusion, and erythema of the affected joints, most commonly the knee joint. While some cases are found with fever and elevated inflammatory markers, clinical presentations may vary [2]. In our case, the patient presented in the emergency department with severe pain, swelling, and warmth of the left knee joint with a moderately restricted range of motion.

Pseudoseptic arthritis is clinically indistinguishable from septic arthritis. Septic arthritis is a potentially fatal condition and presents similarly to pseudoseptic arthritis [8]. Other differential diagnoses besides septic arthritis are Lyme disease, viral arthritis, and reactive arthritis [1]. As there is no specific test to diagnose pseudoseptic arthritis, bedside arthrocentesis with further analysis is indicated to rule out septic arthritis [2]. Both septic arthritis and pseudoseptic arthritis lead to purulent synovial fluid [2]. After arthrocentesis, cloudy appearing synovial fluid was seen in our case. In pseudoseptic arthritis, synovial fluid WBC count was found to be reported from 18,000 to greater than 400,000 [2]. In our case, the synovial fluid WBC count was 89,820. A purulent joint should always be considered septic until the result is negative for culture [7]. Purulent synovial fluid with marked leukocytosis could be indicative of either septic arthritis or pseudoseptic arthritis [2]. The only way to differentiate pseudoseptic arthritis from septic arthritis is by synovial fluid culture and response to steroids or NSAID instead of antibiotics [1,2]. In our case, no clinical improvement after broad-spectrum antibiotics and negative synovial culture ruled out septic arthritis. Thus, antibiotics were discontinued.

Pseudoseptic arthritis is not a self-limiting disease and requires treatment with NSAID, corticosteroid (systemic or intra-articular), and joint fluid aspirations [5,7]. In our case, NSAID was not prescribed due to CKD. Therefore, steroid was initiated, and the patient’s symptoms improved rapidly. This rapid resolution of symptoms is rarely seen with septic arthritis [8]. Timely initiation of NSAID or steroid reduces hospital stay and patients are reported to be discharged after an average of six days of hospitalization [2]. In our case, the patient was discharged on the 7th day of hospitalization.

Pseudoseptic arthritis could be the initial presentation of ankylosing spondylitis [6]. In our case, the patient was found to have typical psoriatic skin lesions on the scalp and back for years, onycholysis of nails, and positive family history of psoriatic arthritis. Along with multiple joint involvements, the patient was diagnosed with psoriatic arthritis. So, in this case, pseudoseptic arthritis is the initial presentation of psoriatic arthritis.

Pseudoseptic arthritis is a recurrent condition and it can lead to joint destruction [9]. Furthermore, underlying psoriatic arthritis in this patient might aggravate the joint condition and result in long-term morbidity. Therefore, the patient was started on leflunomide along with steroid with outpatient Rheumatology follow-up. Though rheumatoid factor was positive in this patient, it can be seen in up to 10% of cases of psoriatic arthritis [10]. Since the clinical features are suggestive of psoriatic arthritis, anti-cyclic citrullinated peptide (CCP) was not done. Though the exact mechanism of pseudoseptic arthritis in the context of psoriatic arthritis is unknown, immunological pathologies might play a role leading to pseudoseptic arthritis.

## Conclusions

Pseudoseptic arthritis is a diagnosis of exclusion. Clinicians should have a high index of suspicion for pseudoseptic arthritis while treating presumptive septic arthritis with a sterile synovial fluid study. Thus, we can avoid unnecessary antibiotic therapy and possible orthopedic joint intervention. Early recognition and initiation of appropriate treatment can hasten recovery and decrease hospital stay. Pseudoseptic arthritis could be the initial presentation of undiagnosed psoriatic arthritis. Further studies might help us to understand better the disease mechanism of pseudoseptic arthritis with underlying psoriatic arthritis.
